# Graphene Oxide/Reduced Graphene Oxide Enhanced Noniridescent Structural Colors Based on Silica Photonic Spray Paints with Improved Mechanical Robustness

**DOI:** 10.3390/nano11040949

**Published:** 2021-04-08

**Authors:** Jiali Yu, Cheng-Hao Lee, Chi-Wai Kan

**Affiliations:** Institute of Textile and Clothing, The Hong Kong Polytechnic University, Hung Hom, Kowloon, Hong Kong SAR 999077, China; scarlett.yu@connect.polyu.hk (J.Y.); chenghao.lee@polyu.edu.hk (C.-H.L.)

**Keywords:** structural color, SiO_2_, nanoparticles, paints, graphene oxide, reduced graphene oxide, spray coating

## Abstract

In contrast to traditional pigment colors, structural colors have developed a great potential in practical applications, thanks to their unique nonfading and color tunable properties; especially amorphous photonic structures with noniridescent structural colors have attracted considerable attention and their applications have expanded to more fields. Herein, graphene oxide (GO) and reduced graphene oxide (RGO) enhanced noniridescent structural colors with excellent mechanical robustness were established by a time-saving approach named spray coating, which allows for rapid fabrication of angular independent structural colors by spraying different photonic spray paints (PSPs) to ensure color multiplicity that was adjusted by the silica nanoparticles (SiO_2_ NPs) sizes onto the substrates. The incorporation of poly(methyl methacrylate-butyl acrylate) (PMB) improved the adhesion existing among SiO_2_ inter-nanoparticles and between SiO_2_ NPs and the substrates, taking advantages of the low glass transition temperature ($T_{g}$) of butyl acrylate derivative polymer and made PMB embedded PSPs coated patterns being imparted with good mechanical robustness and abrasive resistance. The peculiar light adsorption of GO and RGO across visible light spectrum facilitate higher color saturation. The improvement in color saturation of GO and RGO doped PSPs is expected to boost the promising applications in structurally colored paintings, inks and other color-related optical fields.

## 1. Introduction

Noniridescent structural colors from amorphous photonic structures with short-range ordered arrangement based on incoherent light scattering [1,2,3], have attracted much scientific interest in recent years, owing to their superior non-fading, color tunable and angle-independent characteristics [2,4]. The materials with these superior optical performances and quasi-periodic, controlled disordered structures, namely photonic glasses or photonic alloys, have been proposed [5,6,7], different from photonic crystals that have long-order arranged structures by self-assembly [8,9]. Some fabrication approaches including electrophoretic deposition and dip coating, used to create structural-colored materials, seem to be delicate and time-consuming [1,10,11]. However, spray coating [12] is a relatively facile approach for rapid patterning structural colors especially angle-independent noniridescent structural colors as this requires minimal PSPs materials for spraying and is beneficial in mass production. Besides, it is widely accepted that SiO_2_ NPs and its functionalized derivatives to form the building blocks for generating structural colours [13,14,15,16,17]. Kraft paper is a significant cellulose-based renewable material [18], its nonwoven porous structures with large surface areas, hydrophilicity, certain thickness and stiffness make it a good candidate as the substrates for constructing structural colors.

The amorphous photonic structures with certain thicknesses possessing a strong incoherent light scattering in the visible light region results in whitish structural colors. In order to resolve this issue, black substances including carbon black [19,20,21], polydopamine [3,22] in small sizes and quantities have been used to suppress incoherent multiple scattering and improve the color visibility. Advanced carbon materials such as carbon nanoparticles, graphene oxide, graphene possessing exceptional mechanical, electrical, thermal and optical properties [23,24,25,26,27], contributing to the improvement of reflection color and visual appearance [28,29,30]. However, integration of these black materials with the structural color system has not been much studied. Moreover, since the structural stability of the photonic crystal or colloids film is poor due to the high $T_{g}$ of SiO_2_ and weak van der Waal’s forces among the SiO_2_ NPs [31], and the fact that the black dopants cannot contribute to the mechanical robustness of structural color arrays, PMB was regarded as such an additive, benefiting from its relatively low $T_{g}$. The robust PSPs coated patterns are easily fabricated, which means that embedding PMB with SiO_2_ NPs suspension is expected to endow the resulting PSPs coated films with strengthened adhesive performance and abrasion resisting properties.

**Scheme 1:**
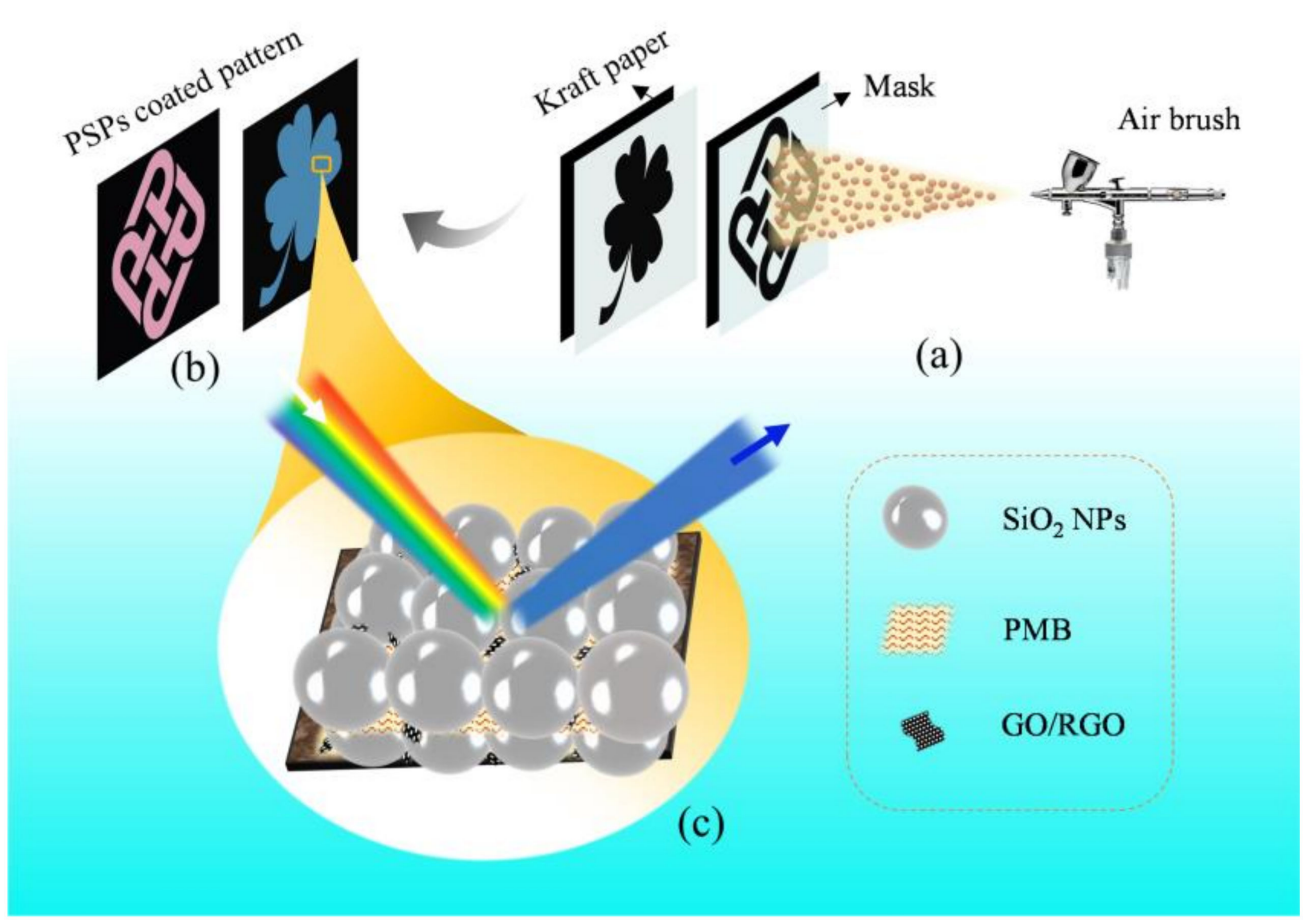
(**a**) Schematic diagram of spray coating process of SiO_2_ PSPs on kraft paper using masks. (**b**) SiO_2_ PSPs coated patterns. (**c**) Model of SiO_2_ PSPs coated patterns with PMB dopants and GO or RGO enhanced noniridescent structural colors.

In this paper, we present structural-colored PSPs on kraft paper substrates produced by spray coating that endows PSPs coated patterns with nearly angle-independent properties and noniridescent structural colors (Scheme 1). By controlling the SiO_2_ NPs sizes, certain ratios of dopants (PMB, GO, RGO) and thicknesses of sprayed PSPs coatings, diverse noniridescent structural colors can be easily realized. PSPs coated structurally colored materials with GO and RGO enhanced colors with excellent structural stability are expected to be desirable in broader applications in the fields of paintings and optical related areas.

## 2. Materials and Methods

### 2.1. Materials and Chemicals

Tetraethyl orthosilicate (TEOS) (reagent grade), ammonia (NH_3_, 25% in H_2_O) and EtOH, 99.9%), sodium dodecyl sulfate (SDS), methyl methacrylate (MMA), butyl acrylate (BA, ≥99%), ammonium persulfate (APS, ≥98.0%, ACS reagent), graphene oxide (4 mg·mL^−1^ dispersion in H_2_O), reduced graphene oxide (powder) were purchased from Sigma-Aldrich (St. Louis, MO, USA). Deionised water was obtained from a Thermo Scientific GenPure Standard Water Purification System (Niederelbert, RP, Germany). GO and RGO were diluted and dispersed in deionized water by ultrasonication and formulated into 0.08 mg·mL^−1^ aqueous solution before using. All chemicals were used as received from their venders without any further purification.

### 2.2. Preparation of PMB

To enhance the structural stability of the noniridescent structural colours, a kind of adhesive was synthesized by the following steps: 5.0 g MMA, 5.0 g BA, 1.0 g SDS, 25.0 g deionized water were sequentially added into a 100 mL double-deck three-necked flask in a water cycle bath that was controlled by Lab Companion RW3-0525 Refrigerated & Heating Bath Circulator, with magnetic stirring at a speed of 500 rpm. After 30 min magnetic mixing at 80 °C, APS solution (0.1 g APS pre-dispersed in 5.0 g deionized water) was slowly added into the mixture. Afterwards, the mixture was stirred vigorously, and the reaction process was still kept at 80 °C under a Nitrogen (N_2_) atmosphere. The polymerization was carried out for 4 h and finally the weight ratio of the obtained PMB solution was 12.66%.

### 2.3. Preparation of SiO_2_, SiO_2_/PMB, SiO_2_/PMB/GO, SiO_2_/PMB/RGO PSPs

Various sizes of SiO_2_ NPs were prepared based on the change of EtOH amount from 110 to 200 mL. Deionized water (15 mL) and TEOS (14 mL) were added under magnetically stirring at 500 rpm into a 250 mL double-deck three-necked flask equipped with a constant temperature water circulation system, followed by addition of 14 mL of ammonia water as a catalyst. After two-hour reaction process at a constant temperature of 60 °C, SiO_2_ NPs in suspension with different sizes were achieved by adjusting the amount of EtOH. The synthesized suspension solution of SiO_2_ NPs was centrifuged and purified by centrifugation at 6000 rpm for 20 min. Then the supernatant solution was discarded, SiO_2_ NPs were dispersed and cleaned by deionized water. The purification step was repeated three times to obtain pure SiO_2_ NPs. SiO_2_ PSPs was obtained by re-dispersing the purified SiO_2_ NPs into deionized water with 10% mass fraction. PMB (5.0% to SiO_2_ NPs) was added into 10% SiO_2_ NPs stock solutions, and thus SiO_2_/PMB PSPs was obtained. In order to explore the GO or RGO effects on structural colors, GO or RGO was mixed with SiO_2_/PMB suspension (5.0% PMB to SiO_2_ NPs) by ultrasonication to prepare SiO_2_/PMB/GO, SiO_2_/PMB/RGO PSPs. Considering the relatively optimal brightness and saturation, GO or RGO (0.01% to SiO_2_ NPs) was added in subsequent experiments.

### 2.4. Fabrication of Noniridescent Structural Colors by Spray Coating

PSPs coated patterns with noniridescent structural colors were facilely established by spray coating process, SiO_2_ PSPs, SiO_2_/PMB PSPs, SiO_2_/PMB/GO PSPs or SiO_2_/PMB/RGO PSPs were sprayed onto kraft paper through hollowed-out patterned polypropylene (PP) masks with continuous spraying every 20 s. Every spaying lasting 10 s was regarded as one layer of PSPs on kraft paper. The distance between the nozzle of air brush and the masks in close proximity to kraft paper substrates was 10 cm. To study SiO_2_ NPs size change with EtOH and clearly observe PMB effect on the structural stability, SiO_2_ photonic crystal films (PCFs) were obtained by following steps: The stock solution of 10% SiO_2_ NPs and PMB (1–10% to SiO_2_ NPs) were used for preparing SiO_2_ PCFs, a droplet of 400 µl was then dropped on preheated cover glasses, followed by 50 min solvents evaporation at 65 °C in an oven, and finally SiO_2_ PCFs with iridescent colors and angle-dependent property were obtained.

### 2.5. Characterization

#### 2.5.1. Microscopic Observation

The microstructure of PMB was detected by JEOL JEM-2011 transmission electron microscope (TEM, JEOL Co., Tokyo, Japan). PMB solution was first diluted by approximately 100 times, 10 nanoparticle solution was then deposited on 400-mesh carbon-coated copper grids, and the sample was completely air-dried prior to TEM observation. Scanning electron microscopy (SEM) images showing surface morphology of the prepared SiO_2_ PCFs with ordered hexagonal-close-packed structures were observed by VEGA3 TESCAN, imaging up to 80,000×, and the micro-images of the PSPs coated patterns with disordered surfaces were performed by JSM-6490 SEM (JEOL Co., Tokyo, Japan). Prior to SEM observation, the non-conductive samples were sputter-coated with a thin gold film to reduce charge effects under micro imaging. The elemental analysis of the prepared samples was achieved by JSM-6490 SEM with an energy-dispersive X-ray spectroscopy (EDS) detector. The scanning probe characterization upon the morphological changes in the surface of SiO_2_ PCFs was analyzed by means of atomic force microscope (AFM) technique (Park systems, XE-100). The scan sizes X and Y were 2 μm with 1.5 Z servo grain at 0.8~1 Hz scan rate.

#### 2.5.2. SiO_2_ NPs Sizes Measurement

The average particle sizes and polydispersity index (Ð) of the as-synthesized SiO_2_ NPs were measured by a Zeta Plus particle size analyzer (Brookhaven Instruments Corp., Holtsville, NY, USA). The particle sizes of dried SiO_2_ NPs were calculated from TEM images by using Nano measurer software, version 1.2, Fudan University, Shanghai, China.

#### 2.5.3. Color Appearance Assessment

Color reflectance and CIE L*a*b* values of the tested samples were analyzed by an SF 650 spectrophotometer (DataColor International, Lawrenceville, NJ, USA) with the D65 diffuse illumination, 8° observing angle and 3 mm/6.6 mm aperture sizes. Wavelengths ranging from 400 to 700 nm were obtained. Each datum was acquired by calculating the average value of four repeated measurements. CIE X and Y coordinates were calculated by ColorTell tool (ColorTell Tech Co., Ltd., Beijing, China) from L*a*b* values that was determined by SF 650 spectrophotometer. Optical images of the structural colour films were captured by a cellular dual 12 MP wide-angle and telephoto cameras under the D65 illuminated light source in which the direction of illumination light is perpendicular to the ceiling of the chamber, and the color reflection images of the samples were obtained at the viewing angle (θ), between the sight line of the camera and the film sample surface. Besides, the light source cabinet was under totally dark environment.

#### 2.5.4. Spectroscopic Measurement

The vibrational spectra of PMB, GO, RGO and PSPs including SiO_2_, SiO_2_/PMB, SiO_2_/PMB/GO and SiO_2_/PMB/RGO were investigated by FTIR and Raman spectroscopy for rapid discrimination of chemical compositions of the prepared samples. FTIR spectrum with absorbance peak was obtained using a Perkin-Elmer Spectrum 100 FTIR spectrometer (Perkin-Elmer Inc., Boston, MA, USA) to analyze the inherent chemical information of PMB, SiO_2_, GO, RGO, SiO_2_/PMB, SiO_2_/PMB/GO and SiO_2_/PMB/RGO PSPs. The samples were scanned from 4000 to 650 cm^−1^ region for 64 scans with a resolution of 4 cm^−1^ at room temperature. Micro-Raman spectrum was measured by Renishaw InVia Micro-Raman Spectroscopy system (Renishaw plc, Gloucestershire, UK) equipped with a confocal optical microscope for fast mapping. Raman wavelength range was between 200 and 2200 nm, the spectral data was collected with resolution of 0.3 cm^−1^, and the stability was <±0.001 cm^−1^. The selected wavenumber ranged from 3200 to 300 cm^−1^, while the tested samples were irradiated with diode laser with excitation wavelength of 785 nm, and the output power of laser was 0.01–5 mW, adjusted by different samples. Raman scatterings from the samples were collected by objective lens with 50× eyepiece for visual observations. A multichannel charge-coupled device (CCD) detector was applied for spectral analyses, and a digital camera with high sensitivity mode was applied for imaging.

#### 2.5.5. Abrasion Test for Mechanical Robustness

The sandpaper abrasion measurement was implemented by adding a 500 g weight on the back surface of a P100 sandpaper that was put face down on the tested samples that were fixed on a flat surface, pulling one end of the sandpaper for 1 s, which leads to 5 cm movement of the weight along with sandpaper sliding over the sample surface. The sandpaper abrasion test was conducted twice for each sample.

## 3. Results and Discussion

### 3.1. Structural Colors Diversity Based on SiO_2_ NPs Sizes

**Figure 1:**
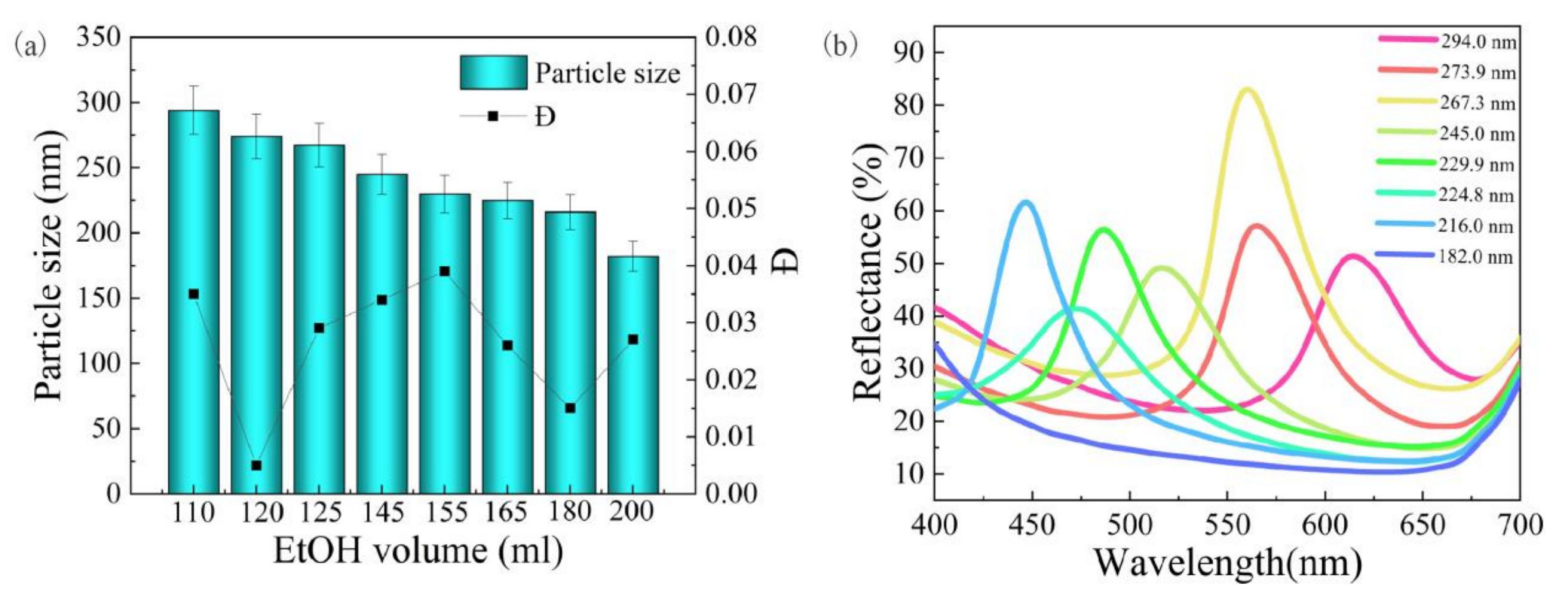
(**a**) Different mean SiO_2_ NPs sizes: 294.0, 273.9, 267.3, 245.0, 229.9, 224.8, 216.0 and 182.0 nm with different Ð values as a function of EtOH amount: 100, 120, 125, 145, 155, 165, 180 and 200 mL respectively. (**b**) The reflectance spectra of SiO_2_ PCFs that were constructed by self-assembled SiO_2_ NPs with controllable sizes in visible light region.

The inverse correlation between SiO_2_ NPs size and the amount of ethanol (EtOH) is revealed intuitively (Figure 1a). Particle sizes of SiO_2_ NPs ranging from 294.0 nm to 182.0 nm were obtained by regulating the amount of EtOH from 110 mL to 200 mL. This study shows the size of the SiO_2_ NPs decreases with the increase of EtOH volume while the amount of TEOS (16 mL), water (15 mL) and ammonia water (14 mL) remain fixed. The reflectance spectrum of the fabricated SiO_2_ PCFs varying from SiO_2_ NPs sizes exhibiting iridescent structural colors on glasses shows conspicuous photonic bandgap displacement and proves a wide range of structural colors across the visible light region from 400 nm to 700 nm (Figure 1b). As the SiO_2_ NPs sizes decrease, the related wavelength of the photonic bandgap has a distinct blue shift. The result is consistent with the theoretical basis of Bragg’s law Equation (1) [32,33], in which the characteristic peak wavelength of diffraction (λ) is directly proportional to the interplanar spacing (d) that is closely correlated to the sizes of self-assembled SiO_2_ NPs,

(1) $$m\lambda =2d\sqrt{n_{eff}{}^{2}-sin_{\varphi }{}^{2}}$$

where m represents the series of diffraction, $n_{eff}$ means the effective refractive index, φ is the angle of the incident light. It is also indicated that the structural color varies with the angle of incident light, resulting in angle-dependent structural colors in terms of SiO_2_ PCFs.

**Figure 2:**
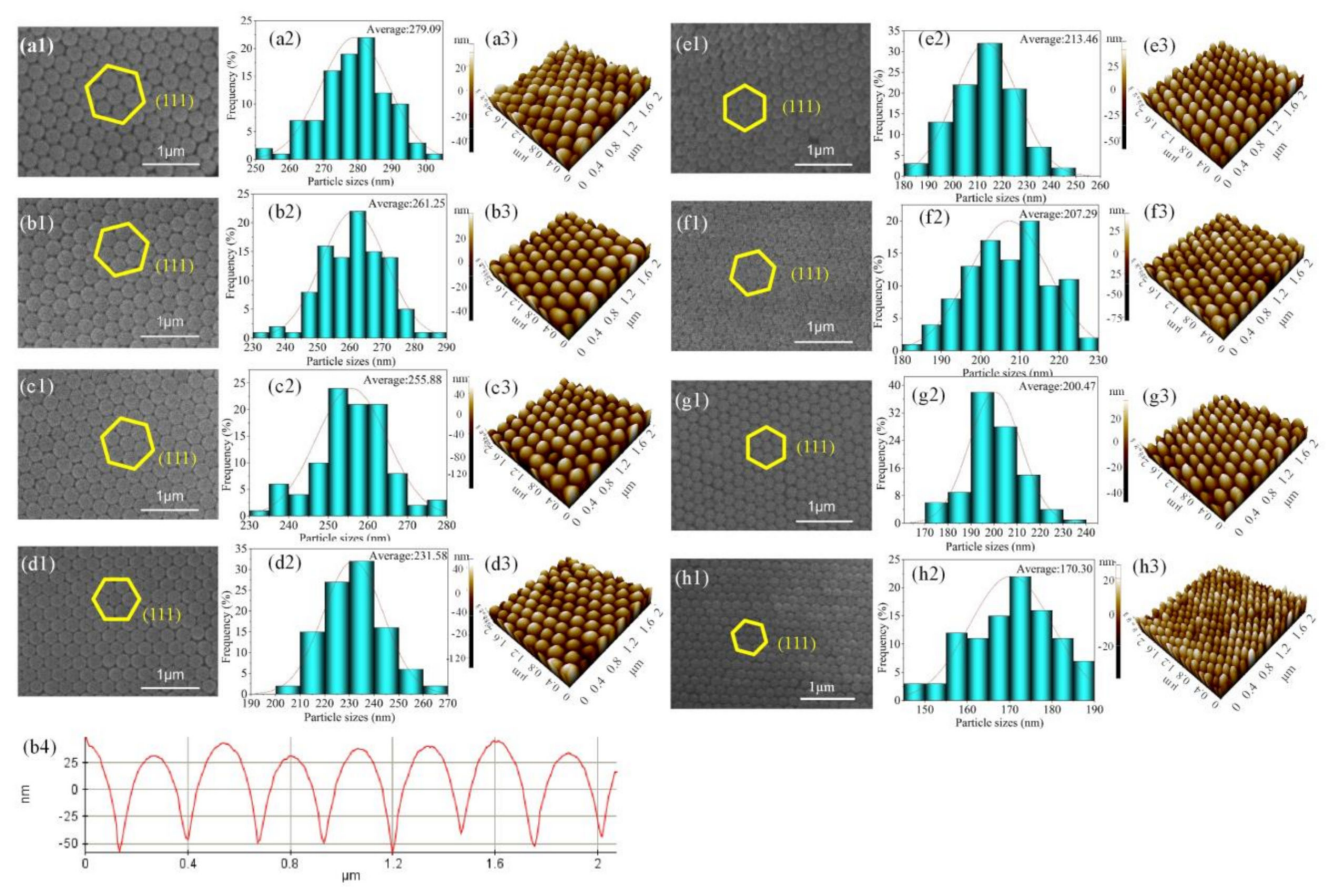
SiO_2_ PCFs with SiO_2_ NPs sizes (**a**) 294.0 nm, (**b**) 273.9 nm, (**c**) 267.3 nm, (**d**) 245.0 nm, (**e**) 229.9 nm, (**f**) 224.8 nm, (**g**) 216.0 nm, (**h**) 182.0 nm measured by Zeta Plus particle size analyser. (**a1**–**h1**) SEM micrographs of SiO_2_ PCFs with honeycomb structures as shown in the areas of yellow frames (magnification: 80,000×). (**a2**–**h2**) histogram of corresponding particle size distribution with different average diameters measured by Nano Measurer software: 279.09 nm, 261.25 nm, 255.88 nm, 231.58 nm, 213.46 nm, 207.29 nm, 200.47 nm, 170.30 nm. The red lines were the curves obtained by Gaussian fitting. (**a3**–**h3**) Three-dimensional (3-D) representations of surface morphology of SiO_2_ NPs measured by AFM showing within close-packed hexagonal cone structures. The measured area is 2 × 2 μm^2^. (**b4**) Section analysis of SiO_2_ NPs (273.9 nm) by atomic force microscopy (AFM).

It was found from SEM observation that SiO_2_ NPs with the spherical shapes and relatively uniform diameters were arranged in hexagonal close packed structures periodically. These honeycomb like crystalline structures of SiO_2_ PCFs were found in the area of yellow boxes as shown in Figure 2a1–h1. The SiO_2_ NPs sizes decreased apparently with the increase of EtOH volume from both SEM (Figure 2a1–h1) and AFM images (Figure 2a3–h3). It coincided well with experimental results as illustrated in Figure 1a. Histograms (Figure 2a2–h2) were used to intuitively analyse SiO_2_ particle size distribution with different average diameters measured from SEM images by Nano Measurer software, the average SiO_2_ NPs sizes were calculated after measurement of the diameters of 100 selected SiO_2_ NPs from the SEM micrographs. It was showed that diameters of each kind of SiO_2_ NPs were normally distributed and comparatively centralized in certain average particle sizes. There was a difference in particle size results measured by Zeta Plus particle size analyser and Nano Measurer software. It was showed that numerical difference of SiO_2_ NPs sizes obtained by Nano Measurer from SEM images was more than 10 nm smaller than that measured by Zeta Plus particle size analyzer. The reason behind this phenomenon could be attributed to the different SiO_2_ NPs states based on different test conditions, in which Brownian dynamics of SiO_2_ NPs existed in diluted aqueous suspension that was used for particle size analysis by Zeta Plus analyzer, while dried SiO_2_ NPs with removal of aqueous media can be regarded as SEM samples, the solvent evaporation shielded SiO_2_ NPs size measurement from the effect of Brownian hydrodynamic interactions, and thus results in smaller nanoparticle sizes. From AFM observation (Figure 2a3–h3), the measurements on the topography of these SiO_2_ NPs further confirmed the occurrence of the expected arrangement consisting of hexagonal arrays on the surface of the SiO_2_ PCFs with few defects, and there was a manifest decrease in particle sizes with EtOH volume increase in accordance with the SEM results. Particle sizes of each sample were estimated to be uniform and had dimensional homogeneity. Moreover, the spherical integrity has been confirmed, however, it is interesting that the shape of SiO_2_ NPs seems to be rugby-like (Figure 2b4), the distortion of the particle shape in AFM images is probably ascribed to image signals derived from the geometrical interaction between the particle surface and the finite size pyramidal-shaped tip. TEM results proved that SiO_2_ NPs are in spherical shapes (Figure S1).

### 3.2. Noniridescent Structural Colours by Spray Coating

**Figure 3:**
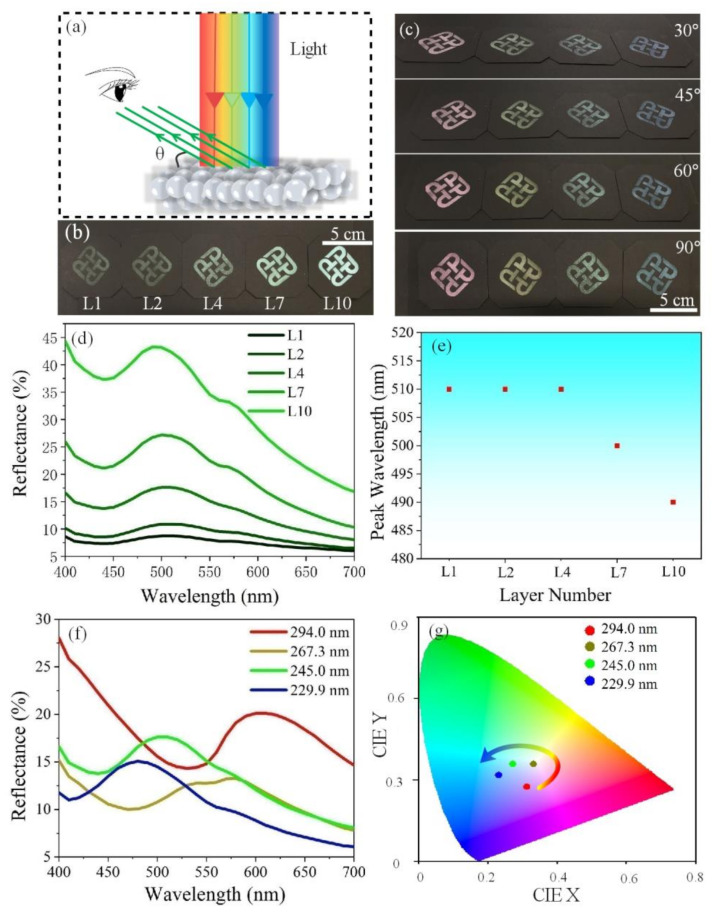
(**a**) Schematic representation of structural color observation with viewing angle (θ). (**b**) Photographs of SiO_2_ PSPs (245 nm) coated PolyU logo patterns with different thicknesses of PSPs (various spraying layers) from 1 to 10 (L1 to L10). (**c**) Photographs of PolyU logo patterns with four structural colors using SiO_2_ PSPs with different SiO_2_ NPs sizes from 294.0 nm, 267.3 nm, 245.0 nm to 229.9 nm at different viewing angles (30°, 45°, 60°, 90°). (**d**,**e**) Reflectance spectra and the corresponding maximum reflection peak wavelength of PSPs coated patterns in (**b**). (**f**) Reflectance spectra of the four structural colors in (**c**). (**g**) The CIE chromaticity diagram of the four structural colors in (**c**).

Color properties of SiO_2_ PSPs coated patterns exhibiting noniridescent structural colors by spray coating method was evaluated (Figure 3). The PSPs coated patterns with 1—10 spraying layers (L1 to L10) were selected as the research objects, in which the selected SiO_2_ NPs is 245 nm (Figure 3b). Spraying 1 or 2 layers of PSPs on the substrates exhibited gloomy and muted structural colors. After spraying reached 4 layers (L4), the structural color tends to be clearer; the unevenness of the structural color is attributed to the shaking of air brush during spray coating process. However, as the spraying layers reached 7 or 10 layers, the structural colors seemed to be whitish. Numerical analysis on reflectance of SiO_2_ PSPs with diverse thickness is shown in Figure 3d. The reflectance peak of L1 and L2 is lower (around 10%) due to the incomplete coverage of SiO_2_ PSPs on black substrate, resulting in absorbance of scattering. As the spraying layers increased to L4, the reflectance peak increased (around 20%), and after further spraying, the peak reflectance reached 28% and 45%, respectively. The results correspond to the observation by naked eye (Figure 3b). We selected PSPs coated patterns with 4 spraying layers (L4) as the following objects to study the angle-independence property of SiO_2_ PSPs coated patterns (Figure 3c,e–g). The observation method is displayed schematically in Figure 3a, in which the viewing angle is θ. Figure 3c proves the slightly angle-dependent property of PSPs coated patterns exhibiting diverse noniridescent structural colours at 30°, 45°, 60° and 90° and the structural colours were hardly changed with the change of viewing angle. The reflectance spectra of these patterns with four distinct structural colors (Figure 3f). It is noticeable that broader photonic bandgap based on coherent reflection is yielded by photonic amorphous structures in short-range order of PSPs coated patterns by spray coating, substantially different from the narrow one in photonic crystalline arrays of SiO_2_ PCFs (Figure 1 and Figure 2). While the sizes of SiO_2_ NPs decreased from 294.0 nm, 267.3 nm, 245.0 nm to 229.9 nm, the photonic bandgap peak wavelength had a similar synchronized decline from 610, 590, 510 to 480 nm. For more visualized observation, these four structural colors were converted into Commission Internationale de l′Eclairage (CIE) chromaticity coordinates (Figure 3g).

### 3.3. Mechanical Robustness Enhancement

**Figure 4:**
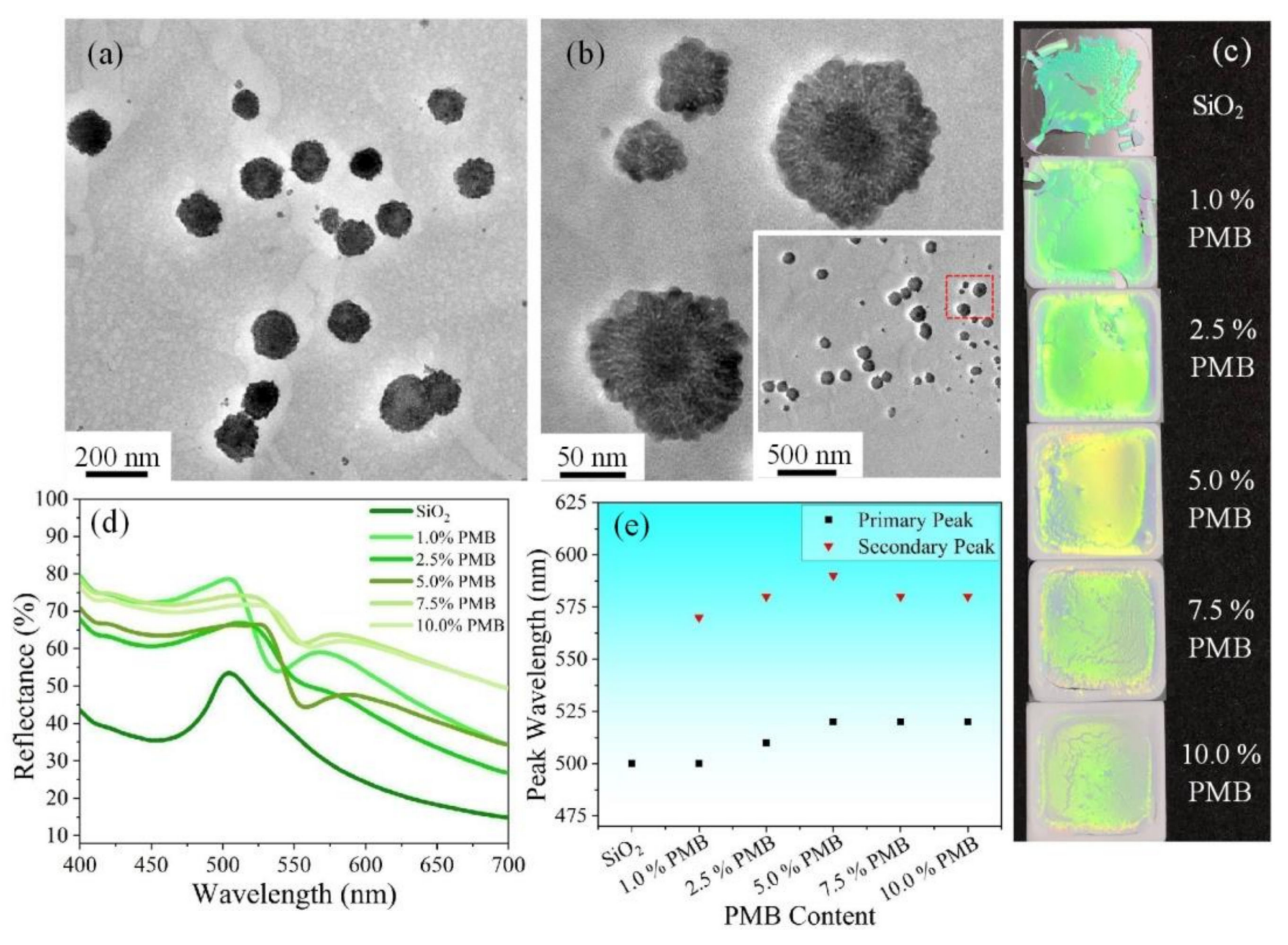
TEM images of poly(methyl methacrylate-butyl acrylate) (PMB) with magnification of (**a**) 12,000×, (**b**) 50,000×, inserted zoom-out image: 8000×. (**c**) Photos of SiO_2_ PCFs with different concentrations of PMB on glasses. (**d**) The related reflectance of (**c**). (**e**) The peak photonic bandgap wavelength of (**d**).

Due to the pure SiO_2_ PCFs or PSPs suffering from unstable structural colours and poor mechanical strength with the substrates, PMB was introduced as a kind of binder to improve structural stability. To manifestly investigate the adhesion function of PMB on SiO_2_ PCFs, photonic crystal films with structural colors were fabricated on glass slide substrates with smooth and frictionless surfaces. The structures of PMB copolymer were displayed as the TEM observation from Figure 4a,b. Poly(butyl acrylate) occupied the positions between the adjacent SiO_2_ NPs and provided the essential adhesion among them. Differential scanning calorimetry (DSC) result indicated low $T_{g}$ (around −50 °C) of PBA and it showed the low glass transition temperature around −50 °C to 0 °C, indicating good elasticity of PMB. The existence of PMMA was also proved because $T_{g}$ around 95 °C was associated with PMMA (Figure S2). Brilliant structural colours (Figure 4c) were generated from long-ordered crystalline structures of SiO_2_ PCFs owing to the narrow and sharp photonic bandgap (Figure 1b). It can be obviously seen from Figure 4c that bare SiO_2_ PCFs was fragile and easily peeled off from the substrate due to its high $T_{g}$, and this phenomenon is also ascribed to the weak inter-interactions among SiO_2_ photonic crystal building blocks as well as interactions between SiO_2_ PCFs and the substrate [34]. With the increase of PMB amount, there were hardly any cracks and disintegrating slags of SiO_2_ PCFs which confirms that PMB made SiO_2_ PCFs that made it having mechanically robust and it was strongly bonded to the substrates, thanks to the extremely low $T_{g}$ of the polymer synthesised by BA monomer [35]. Nonetheless, too much PMB (7.5% and 10.0%) resulted in many cracks of SiO_2_ PCFs because of PMB being the obstacle in self-assembly of SiO_2_ NPs with the removal of solvent by evaporation. It is noticed that the structural colors have an obvious red shift with the increase of PMB content (Figure 4c), which is verified by the reflectance in Figure 4d. Evidently, the primary peak wavelength is enlarged from 500 nm, 510 to 520 nm as the PMB content increased from 1.0% to 10.0%, and it represents the red shift of color (Figure 4e). Interestingly, reflectance of bare SiO_2_ PCFs shows a single peak, however, double peak of reflectance appears with the addition of PMB (Figure 4d,e). Taking SiO_2_ PCFs with 5.0% PMB as an example, the corresponding structural color becomes yellowish on blending green (primary peak: 520 nm) and reddish orange (secondary peak: 590 nm). It can be explained that the inclusion of PMB changed the effective refractive index (n) in photonic crystal lattice as illustrated in Equation (1).

### 3.4. Inclusion of GO and RGO into PSPs Coated Patterns

**Figure 5:**
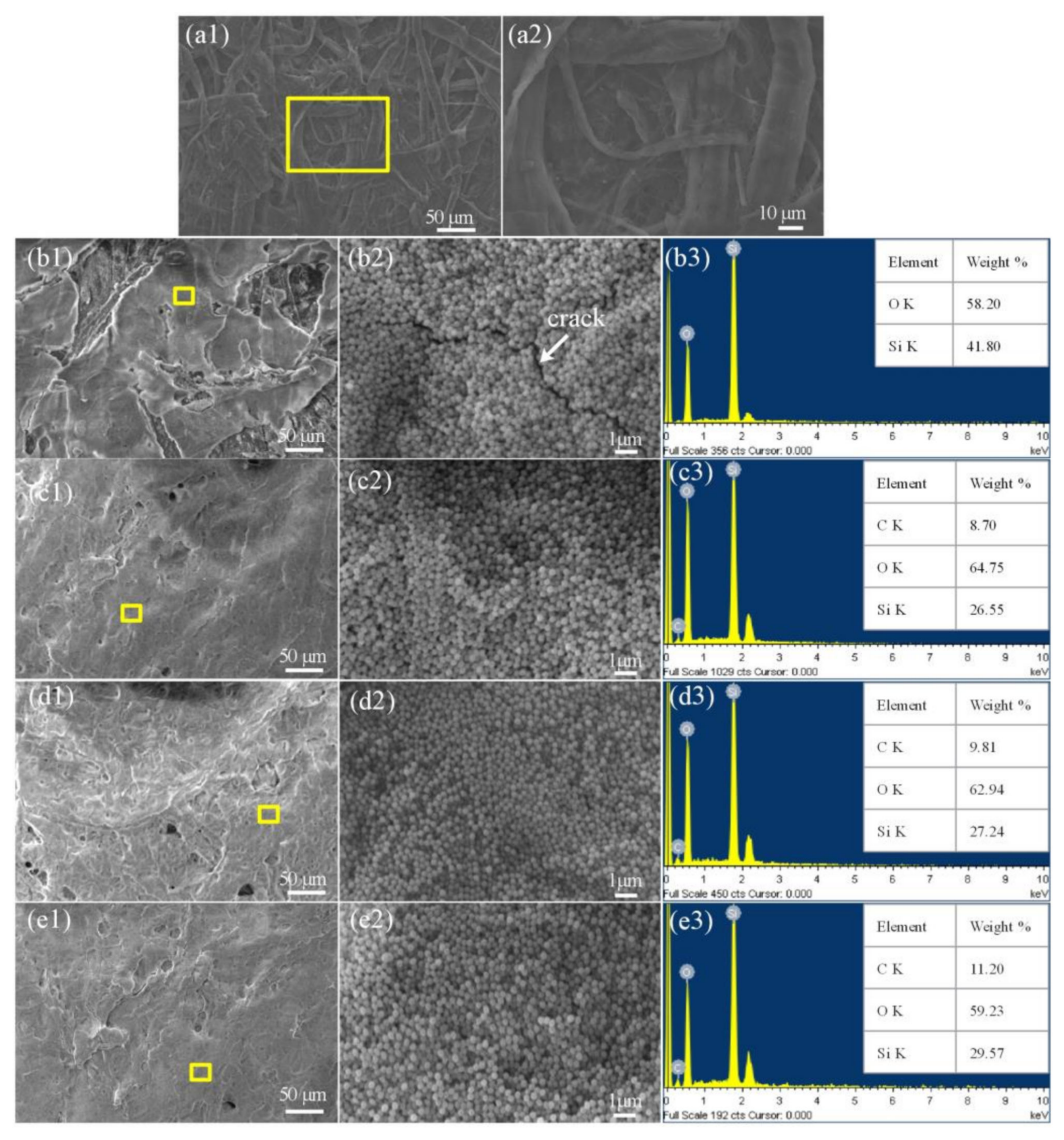
(**a**) Surface morphology of kraft paper from SEM observation with magnification of (**a1**) 300× and (**a2**) 1000×. (**b**–**e**) SiO_2_, SiO_2_/PMB, SiO_2_/PMB/GO and SiO_2_/PMB/RGO PSPs, respectively. (**b1**–**e1**,**b2**–**e2**) surface morphology with magnification of (**b1**–**e1**) 300× and (**b2**–**e2**) 10,000×. (**b2**–**e2**) represents the zoom-in area of the yellow frame in (**b1**–**e1**). (**b3**–**e3**) Elemental analysis (C, O, Si) of SiO_2_, SiO_2_/PMB, SiO_2_/PMB/GO and SiO_2_/PMB/RGO PSPs.

Since noniridescent structural colors prepared by spray coating method are relatively pale and whitish compared to those generated from PCFs due to the strong incoherent scattering that dilutes the light from photonic bandgap [36], black substances (GO or RGO) with light scattering absorbing ability are introduced in colloidal nanoparticle suspension to improve the color saturation and contrast. Figure 5a1,a2 shows the nanostructures of kraft paper having many openings, holes with large surface contact area, and intrinsic hydrophilicity of cellulosic component are conducive to infiltration of sprayed PSPs and rapid evaporation of water medium during spray coating process. Unlike the crystalline structures of PCFs (Figure 2), photonic amorphous structures existed in PSPs coated patterns created by spray coating as Figure 5b2–e2 shows. Dielectric spherical nanoparticles can possess strong scattering resonances based on Mie theory [37]. Such disordered structures with no long-range order impede light interferences but increase the scattering effect, in which the Mie resonances in dielectric nanoparticles (SiO_2_ NPs) with high refractive index in the several hundred-nanometer regions contribute to generate structural color [33,38]. Figure 5b1,b2 proves that pure SiO_2_ PSPs coated film is vulnerable to cracks on a large scale, while the crack-free surface and continuity of amorphous structures about SiO_2_/PMB PSPs coated films is convincing (Figure 5c1,c2), which corresponds to the investigation in Figure 4c. EDS analysis indicates the existence of PMB with the increase of O weight percent and the appearance of C element from (Figure 5c3). The inclusion of well-dispersed GO and RGO in small nanoscale did not alter the crack-free surface of SiO_2_/PMB/GO and SiO_2_/PMB/RGO coated patterns (Figure 5d2,e2). It can be seen from Figure 5d3,e3 that the decrease of O weight ratio from 62.94% to 59.23% validates the fact that RGO has been reduced from GO before use.

**Figure 6:**
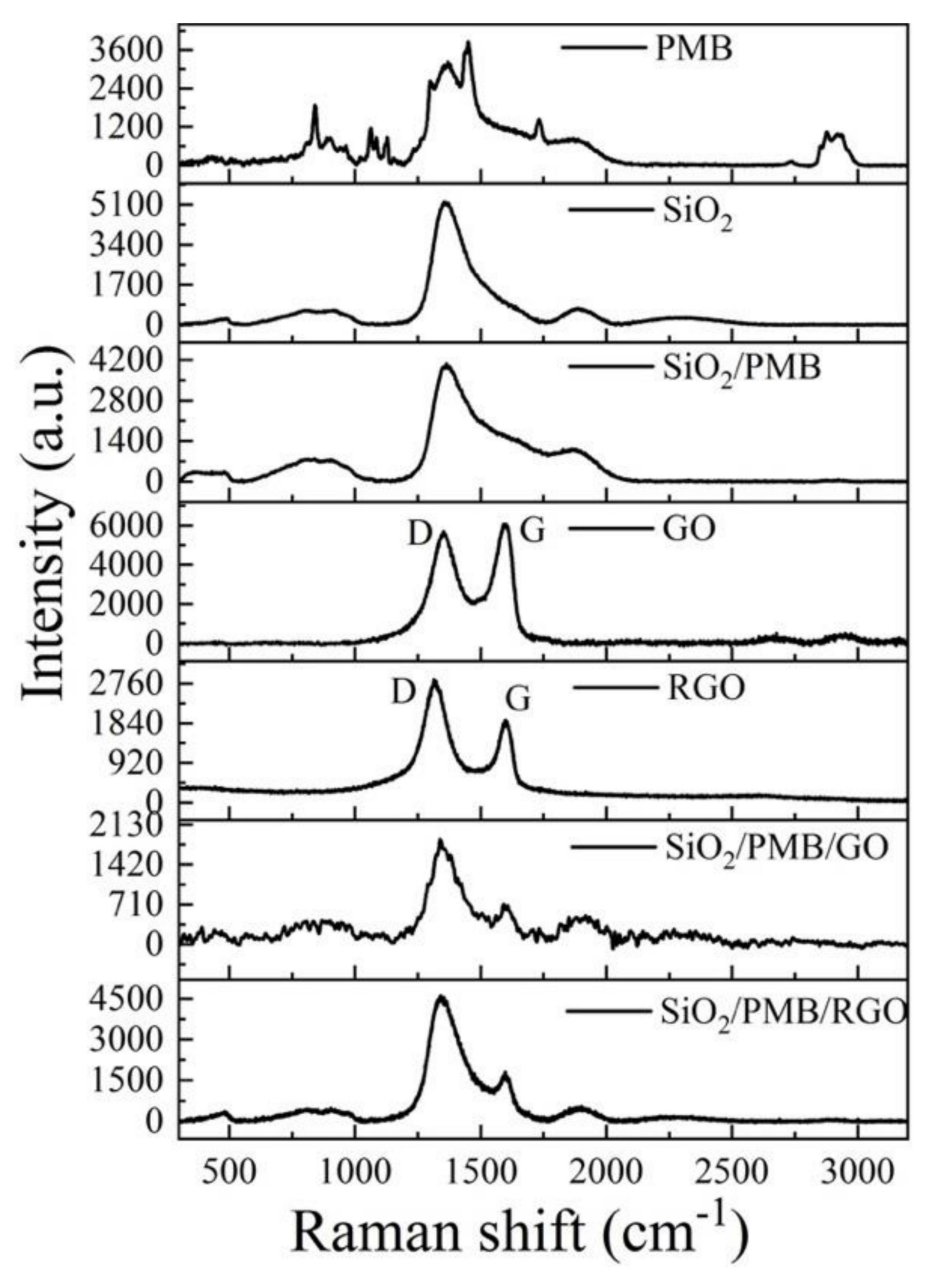
Raman spectrum of PMB, graphene oxide (GO), reduced graphene oxide (RGO) and photonic spray paints (PSPs) including SiO_2_, SiO_2_/PMB, SiO_2_/PMB/GO and SiO_2_/PMB/RGO.

The band profiles of PMB, GO, RGO and SiO_2_, SiO_2_/PMB, SiO_2_/PMB/GO, SiO_2_/PMB/RGO PSPs were performed experimentally by Fourier transform infrared (FTIR) spectrum (Figure S3) and Raman spectra (Figure 6). In FTIR analysis, it is confirmed that the peak at 1731 cm^−1^ belongs to C=O stretching vibration, while the characteristic peak at 1158 cm^−1^ is assigned to C–O–C stretching vibration of the MMA and BA repeating units [39]. The corresponding characteristic peak in transmittance spectra of SiO_2_/PMB PSPs shows PMB with small amounts existed in SiO_2_/PMB PSPs. From Figure S3c, broad band of GO transmittance spectra from 3500 to 2500 cm^−1^ refers to hydrogen bonds of carboxyl COOH groups [40], 3400 cm^−1^ is related to the stretching of hydroxyl group mainly as a result of the presence of absorbed water molecules and alcohol group [41], the peak at 1225 cm^−1^ is attributed to C–OH stretching of alcohol group, C=O carbonyl stretching appeared at 1718 cm^−1^ [42], 1053 cm^−1^ corresponds to C–O group [43]. RGO was obtained by reduction of GO, and thus the related hydroxyl groups are hardly seen in the spectra of RGO; the RGO characteristic peaks are not obvious. The characteristic peaks of GO and RGO are also not apparent in the FTIR spectra of SiO_2_/PMB/GO, SiO_2_/PMB/RGO PSPs owing to the addition of GO and RGO in small amounts (Figure S3d). To further demonstrate the presence of GO and RGO in SiO_2_/PMB/GO, SiO_2_/PMB/RGO PSPs, Raman spectroscopy was performed on PMB, GO, RGO and SiO_2_, SiO_2_/PMB, SiO_2_/PMB/GO, SiO_2_/PMB/RGO PSPs to analyze the interface interaction between nanocomposites and structural changes, the main characteristic peaks of these samples are shown in Figure 6. Two prominent bands of GO at 1354 cm^−1^ (D band) and 1600 cm^−1^ (G band) shifted to 1314 cm^−1^ (D band) and 1597 cm^−1^ (G band) for RGO after reduction. The relative intensity ratio ($I_{D}/I_{G}$) obviously increases from GO to RGO, which indicates more defects generated for RGO after reduction reaction. Analogous experiment has been conducted by Sharma et al. [42]. Raman spectra of SiO_2_/PMB/GO and SiO_2_/PMB/RGO PSPs both show G band, indicating the existence of GO and RGO, respectively.

### 3.5. Mechanical Robustness Measurement

**Figure 7:**
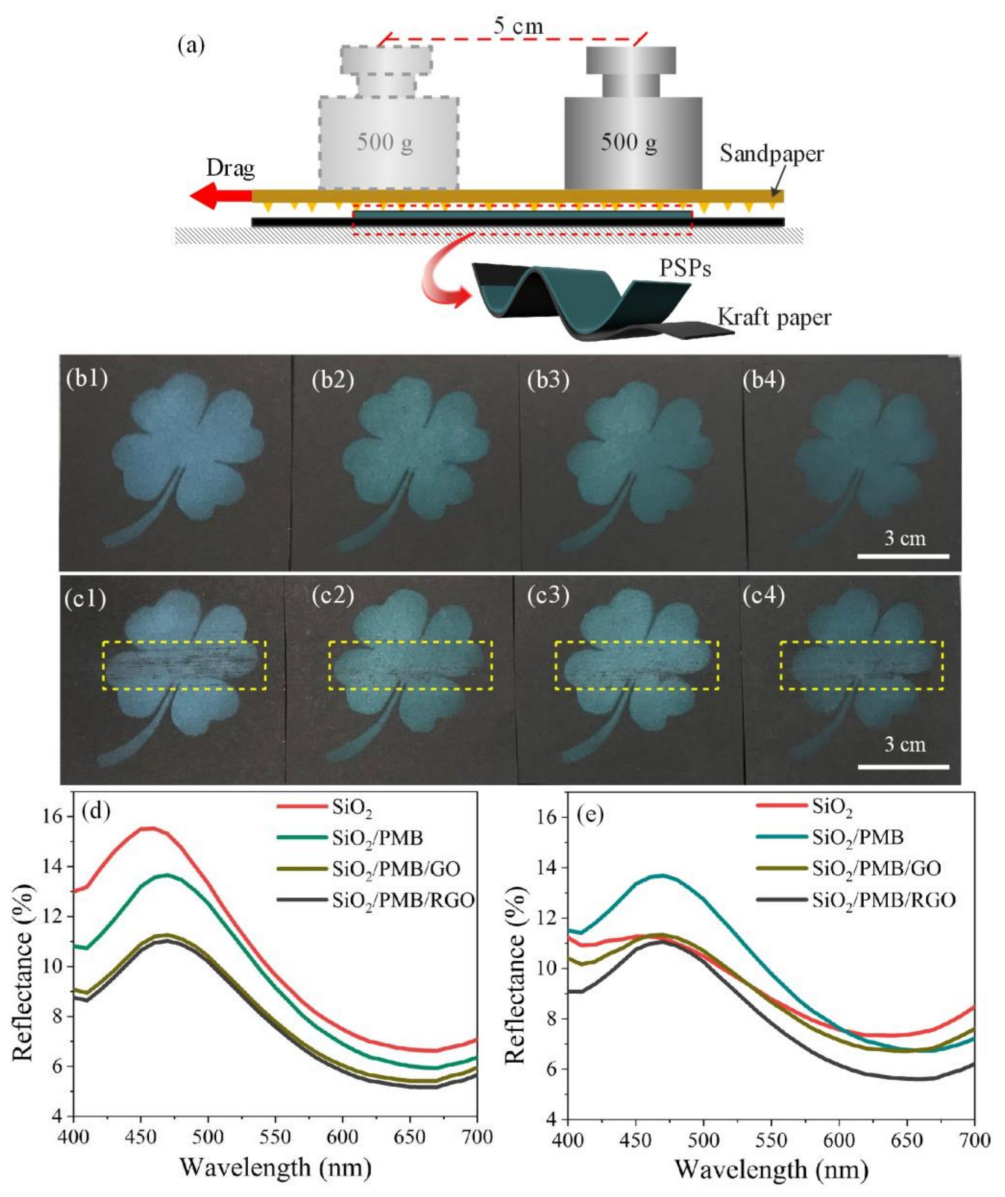
(**a**) Schematic illustration of abrasion test. (**b**,**c**) PSPs coated four-leaf clover patterns (**b**) before and (**c**) after abrasion, (**b1**,**c1**) SiO_2_, (**b2**,**c2**) SiO_2_/PMB, (**b3**,**c3**) SiO_2_/PMB/GO and (**b4**,**c4**) SiO_2_/PMB/RGO (SiO_2_ NPs size: 215.6 nm). The yellow boxes represent the abrasion areas. (**d**,**e**) Reflectance spectra of (**b**) and (**c**), respectively.

Abrasion repellence is another area to expand the wide applications of the PSPs coated paintings for goods preservation. In order to verify whether mechanical robustness of PSPs coated patterns by spray coating is enhanced, we conducted an abrasion test for PSPs coated four-leaf clover patterns. SiO_2_ PSPs coated pattern produced color whitening problem (Figure 7b1), however, color saturation was enhanced with inclusion of GO and RGO (Figure 7b3,b4). The corresponding reflectance (Figure 7d) confirms the decrease of reflectance peak after addition of GO and RGO, mainly thanks to these black dopants that improve the absorbance of light scattering. Upon application of mechanical forces, pure SiO_2_ PSPs coated patterns showed more defects and had more leakage of SiO_2_ NPs after abrasion (Figure 7b,c), which is consistent with the findings of SiO_2_ PCFs (Figure 4). Moreover, as Figure 7d,e displays, the corresponding peak reflectance of SiO_2_ PSPs coated patterns decreased sharply from 15.5% to 11.2% owing to the reveal of black kraft paper substrate, while other SiO_2_/PMB, SiO_2_/PMB/GO and SiO_2_/PMB/RGO coated patterns maintained their structural colors with few defects under friction due to PMB. It is worth nothing that red shift also appeared in SiO_2_/PMB, SiO_2_/PMB/GO and SiO_2_/PMB/RGO coated patterns (Figure 7b2–b4) compared with pure SiO_2_ PSPs coated pattern (Figure 7b1), and the structural colors collaboratively turned from bluish violet to dark yellowish green. The occurrence of red shift is mainly due to addition of PMB polymer adhesive that increased the effective refractive index [31], resulting in the increase of photonic bandgap wavelength. This result is in accord with the investigation that the structural colour change occurred in PCFs with and without PMB (Figure 4c). Correspondingly, the peak reflectance shifted from 460 nm (SiO_2_ PSPs) to 470 nm (SiO_2_/PMB PSPs), the peak reflectance of SiO_2_/PMB/GO and SiO_2_/PMB/RGO coated patterns had no significant change in contrast to SiO_2_/PMB PSPs coated one. Nevertheless, colour saturation of the structural colors was improved with the addition of GO and RGO as can be seen from Figure 7b1–b4.

## 4. Conclusions

In summary, we have demonstrated that GO and RGO enhanced noniridescent structural colors with mechanical robustness were facilely established by spray coating method. SiO_2_ PSPs coated patterns with SiO_2_ NPs of different particle sizes possess various noniridescent structural colors. Since pure SiO_2_ photonic films suffer from relatively poor structural robustness and unstable structural colors, mechanical robustness of SiO_2_ PCFs on a smooth glass substrate with and without PMB additive has been studied to clearly manifest the adhesive property of PMB. The mechanical stability of SiO_2_, SiO_2_/PMB, SiO_2_/PMB/GO, SiO_2_/PMB/RGO PSPs coated patterns has also been examined by the test of sandpaper abrasion, the four-leaf clover patterns with the addition of PMB exhibited better abrasion repellence. The inclusion of GO and RGO improved the color saturation of PSPs coated patterns and helped avoid whitish colors arisen from pure SiO_2_ PSPs. This study provides a feasible approach for quickly constructing noniridescent structural colors and has potential practical applications in painting, optical displays.

## Data Availability

Data is contained within the article.

## Supplementary Materials

The following are available online at https://www.mdpi.com/article/10.3390/nano11040949/s1, Figure S1: TEM images of SiO_2_ nanoparticles with particle sizes of 294.0 nm, scale bars are (a) 100 nm, (b) 1 μm and (c) 100 nm, respectively. (d) The corresponding particle size distribution from TEM image measured by Nano Measurer software, Figure S2: DSC thermogram of PMB, Figure S3: FTIR spectrum of (a) PMB, (b) SiO_2_ and SiO_2_/PMB PSPs, (c) GO, RGO, (d) SiO_2_/PMB/GO and SiO_2_/PMB/RGO PSPs.
