# Supplementary material for: Graphene Oxide/Reduced Graphene Oxide Enhanced Noniridescent Structural Colors Based on Silica Photonic Spray Paints with Improved Mechanical Robustness
Source: Nanomaterials (Basel). 2021 Apr 8;11(4):949. doi: 10.3390/nano11040949 (PMC8068252; doi:10.3390/nano11040949)
Supplement: Supplementary file 1 [file nanomaterials-11-00949-s001.pdf]

Supporting Information

Graphene Oxide/Reduced Graphene Oxide Enhanced Noniridescent Structural Colors Based on Silica Photonic Spray Paints with Improved Mechanical Robustness

Jiali Yu, Cheng-Hao Lee and Chi-wai Kan *

Institute of Textile and Clothing, The Hong Kong Polytechnic University, Hung Hom, Kowloon, Hong Kong SAR 999077, China; scarlett.yu@connect.polyu.hk (J.Y.); chenghao.lee@polyu.edu.hk (C.-H.L.)
* Correspondence: kan.chi.wai@polyu.edu.hk; Tel.: +852-27666531

Citation: Yu, J.; Lee, C-.H.; Kan, C.-w. Graphene Oxide/Reduced Graphene Oxide Enhanced Noniridescent Structural Colours Based on Silica Photonic Spray Paints with Improved Mechanical Robustness. *Nanomaterials* **2021**, *11*, 949. https://doi.org/10.3390/nano11040949

Academic Editor: Gwan-Hyoung Lee

Received: 05 March 2021
Accepted: 06 April 2021
Published: 08 April 2021

Publisher's Note: MDPI stays neutral with regard to jurisdictional claims in published maps and institutional affiliations.

Copyright: © 2021 by the authors. Submitted for possible open access publication under the terms and conditions of the Creative Commons Attribution (CC BY) license (http://creativecommons.org/licenses/by/4.0/).

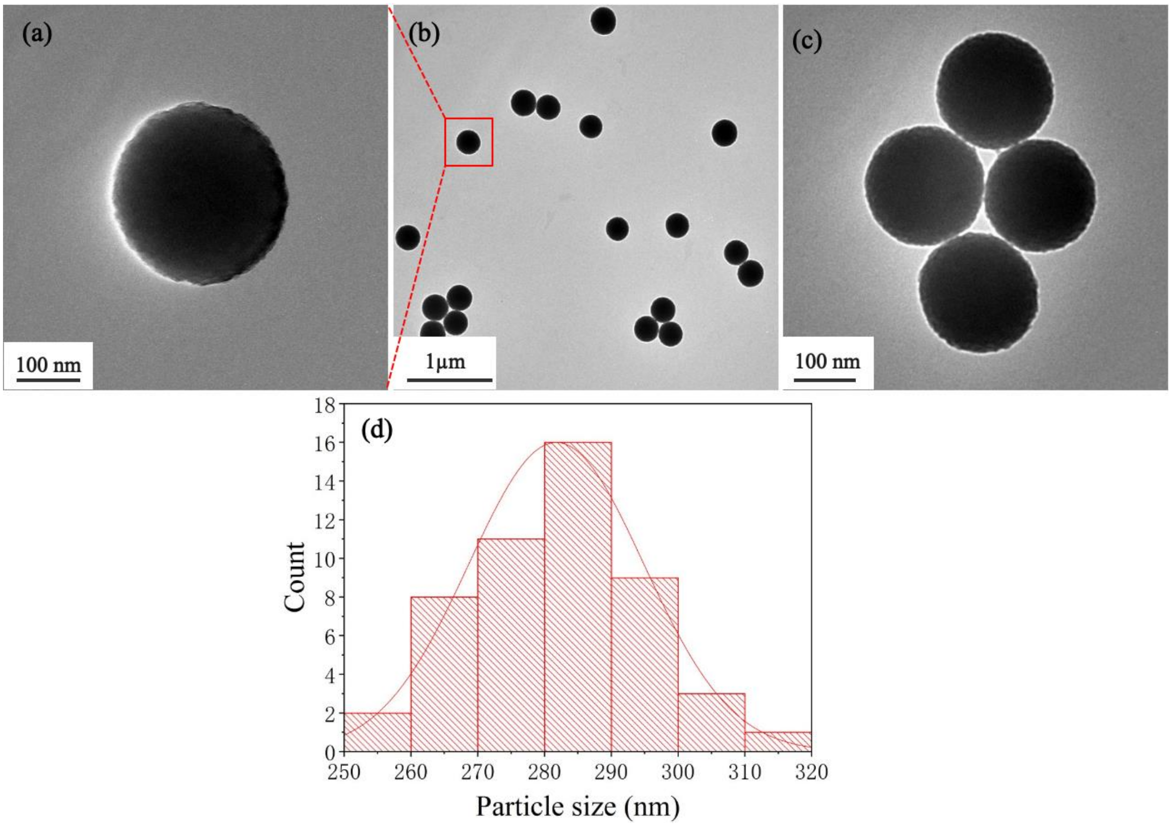

Figure S1. TEM images of SiO_2 nanoparticles with particle sizes of 294.0 nm, scale bars are (**a**) 100 nm, (**b**) 1 μm and (**c**) 100 nm, respectively. (**d**) The corresponding particle size distribution from TEM image measured by Nano Measurer software.

Differential scanning calorimetry (DSC) was performed on a PerkinElmer DSC800 instrument under a dry nitrogen atmosphere. Approximately 7.5mg of PMB was used, and it was heated at a heating rate of 10 °C/min from -65°C to 125°C.

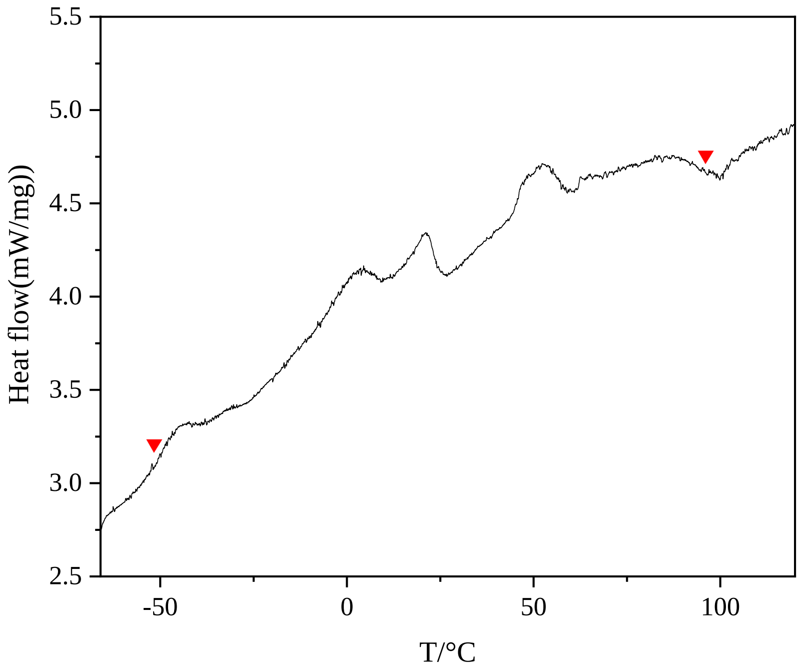

Figure S2. DSC thermogram of PMB.

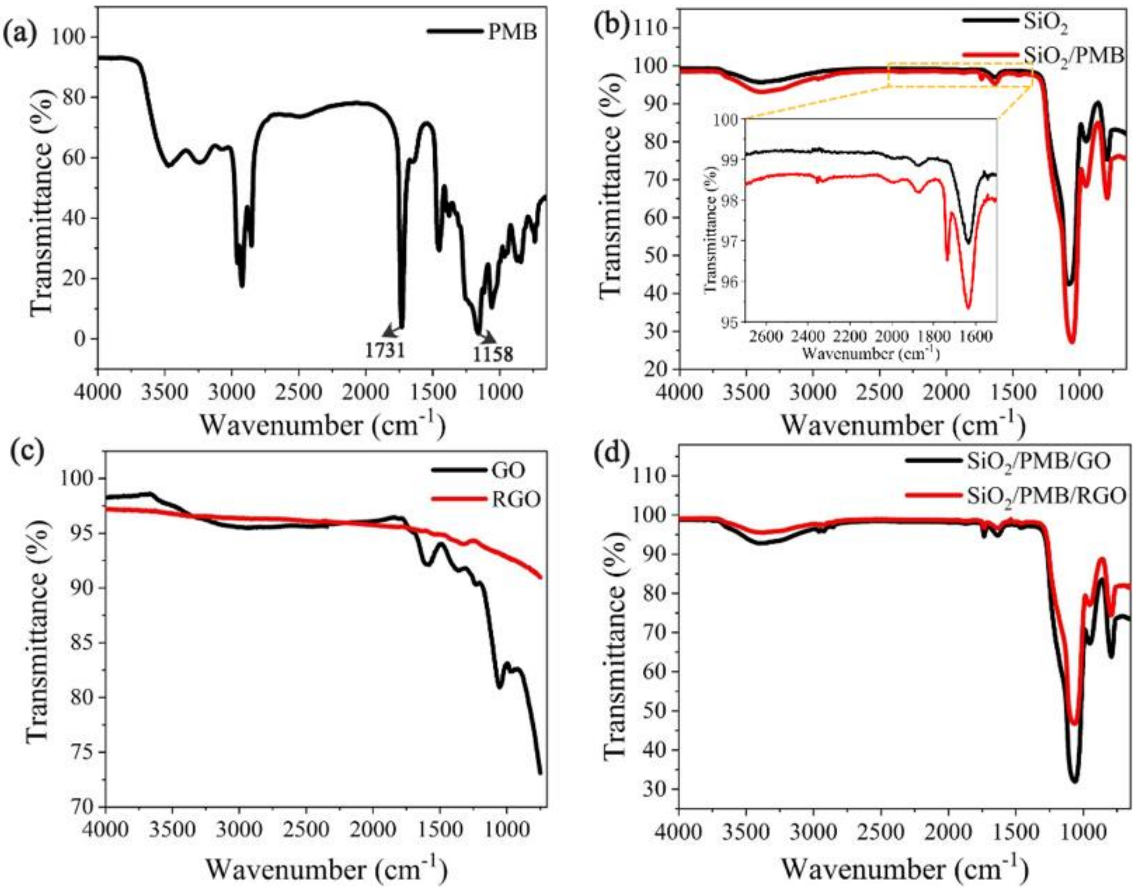

Figure S3. FTIR spectrum of (**a**) PMB, (**b**) SiO_2 and SiO_2/PMB PSPs, (**c**) GO, RGO, (**d**) SiO_2/PMB/GO and SiO_2/PMB/RGO PSPs.
